# Editorial: Exploring novel approaches to small molecule kinase inhibitors in cancer treatment

**DOI:** 10.3389/fchem.2025.1559849

**Published:** 2025-02-03

**Authors:** Anna Carbone, Rafael M. Couñago, Cristian O. Salas, Alessandra Moltalbano

**Affiliations:** ^1^ Department of Pharmacy (DIFAR), University of Genoa, Genoa, Italy; ^2^ Structural Genomics Consortium and Division of Chemical Biology and Medicinal Chemistry, UNC Eshelman School of Pharmacy, University of North Carolina, Chapel Hill, NC, United States; ^3^ Department of Organic Chemistry, Faculty of Chemistry and Pharmacy, Pontificia Universidad Católica de Chile, Santiago, Chile; ^4^ Department of Biological, Chemical and Pharmaceutical Sciences and Technologies (STEBICEF), University of Palermo, Palermo, Italy

**Keywords:** cancer, kinases, kinase inhibitors, small molecules, antitumor agents

The dysregulation of protein kinase activity plays a pivotal role in the pathogenesis of several diseases, including cancer. Therefore, protein kinases have become some of the most important drug targets for antitumor therapy in the 21st century. Approximately 538 known kinases are encoded by the human genome and regulate several biological and cellular processes. The success of imatinib, the first oral tyrosine kinase inhibitor approved by the Federal Drug Administration (FDA) in 2001 for chronic myelogenous leukemia, promoted the extensive use of kinase inhibitors (KIs) as antiproliferative agents (Cohen et al., 2021; Roskoski, 2024). To date, 84 small molecule KIs have been approved by the FDA and 180 drugs are in clinical trials worldwide. Notably, 70 of the FDA-approved KIs are used in targeted cancer therapies (Roskoski, 2024). However, only approximately 20% of the kinome has been targeted so far, underscoring the potential of studying additional kinases to uncover novel treatment options.

Most clinically used KIs act by blocking ATP binding site of active (type I) or inactive (type II) kinases. Beyond these, four additional classes of KIs have been identified: type III and type IV inhibitors, which are allosteric inhibitors targeting sites adjacent to or distant from the ATP-binding pocket, respectively; type V inhibitors, which are bivalent compounds that interact with two distinct regions of the protein kinase domain; and type VI, which are covalent inhibitors (Li et al., 2024). Despite their undoubted advantages, such as reduced side effects compared to traditional chemotherapy and convenient oral administration, KIs face significant limitations. Key challenges include the emergence of acquired drug resistance, occurrence of undesired side effects, and suboptimal pharmacokinetic profiles. These represent unmet clinical needs that need to be overcome (Li et al., 2024). Recently, kinase degraders and protein kinase interactors have emerged as novel strategies for targeting protein kinases (Yu et al., 2021; Klein, 2023; Zerihun et al., 2023). These emerging approaches pave the way for targeting potential new therapeutic targets and overcoming drug-resistant mutations.

Recent advances in the use of kinase inhibitors have been described in this Research Topic, which collected three research articles and a review, received from China, Morocco, Egypt, and Saudi Arabia [https://www.frontiersin.org/research-topics/63906/exploring-novel-approaches-to-small-molecule-kinase-inhibitors-in-cancer-treatment].

In their study, Mohamed et al. reported the design and synthesis of a novel series of quinazoline-4-one/1,2,4-oxadiazole hybrid derivatives. These compounds proved to act as dual inhibitors of EGFR and BRAF^V600E^ with IC_50_ values at the nanomolar level. Further investigation revealed promising activity against the mutant form EGFR^T790M^ with IC_50_ values (15–10 nM) comparable to the reference compound Osimertinib (IC_50_ 8 nM). The putative binding interactions with the key amino acid residues within the active site of the target proteins have been also investigated, thus confirming the potentialities of this class of compounds.

Computational studies are valuable tool for the identification of potential drug candidates. Alsfouk et al. employed a computer-assisted design strategy to identify potential CDK9 and CYP3A4 inhibitors by using a predictive QSAR model and *in silico* synthesis. Molecular docking studies revealed extensive and stable non-covalent interactions between the designed compounds and both targets. In particular, strong H-bond and pi-alkyl interactions were crucial for the compounds’ binding affinity. Molecular dynamics simulations and MM/PBSA analysis further validated the strong interactions between the designed compounds and their targets, sowing the seeds for the development of innovative and effective CDK9 inhibitors.

Similarly, Faris et al. conducted a computational investigation to identify new JAK3 inhibitors. They designed 41 pyrazolo-pyrimidine derivatives bearing an acrylamide group capable of binding the key Cys909 residue in JAK3’s active site. Using covalent docking, ADMET (Absorption, Distribution, Metabolism, Excretion, Toxicity) analysis, molecular dynamics modeling, and MM/GBSA (Molecular Mechanics Generalized Born Surface Area) binding free energy techniques, investigated the potentialities of these molecules have been investigated. While the study focused on the identification of JAK3 inhibitors for treating rheumatoid arthritis, the critical role of JAK3 in the maturation of white blood cells, including T lymphocytes, B lymphocytes, and natural killer cells, makes it a promising target for hematological malignancies (Uckun et al., 2007; Liongue et al., 2024). The results of this study could be a valuable contribution to the development of novel anti-cancer agents.

In their review, Zhang et al. provided an in-depth analysis of MET structure and its key role in non-small cell lung cancer (NSCLS). The MET gene is a crucial oncogenic driver in NSCLC, representing a promising therapeutic target for NSCLC treatment. Several MET small molecule inhibitors, such as capmatinib, savolitinib, and tepotinib, have been approved by the FDA for treating patients with metastatic NSCLC harbouring MET gene mutations. In this context, gave an overview of the three classes of MET inhibitors currently developed and highlighted the mechanisms of acquired resistance to these therapies.

Each contribution to this Research Topic reports different approaches to explore the potential of KIs for cancer treatment, offering new data to the scientific community.
